# Measuring Forgiveness: Psychometric Properties of the Heartland Forgiveness Scale in the Spanish Population

**DOI:** 10.3390/ijerph18010045

**Published:** 2020-12-23

**Authors:** Karla Gallo-Giunzioni, María Prieto-Ursúa, Cristina Fernández-Belinchón, Octavio Luque-Reca

**Affiliations:** 1 Faculty of Education and Psychology, Universidad Francisco de Vitoria, 28223 Pozuelo de Alarcón, Spain; karla.gallo@ufv.es; 2 Department of Psychology, Faculty of Social and Human Sciences, Universidad Pontificia Comillas, 28049 Madrid, Spain; mprieto@comillas.edu; 3 Servicio de Urgencias Médicas de Madrid (SUMMA 112), 28045 Madrid, Spain; cfbelinchon@salud.madrid.org; 4 Department of Psychology, Universidad Rey Juan Carlos, 28933 Madrid, Spain

**Keywords:** instrument, factor structure, brief measure, assessment, Spanish, adults, dispositional forgiveness, validation

## Abstract

Given the scarcity of instruments in Spanish to measure forgiveness, two studies were conducted in this population to obtain validity evidence of the Heartland Forgiveness Scale (HFS), an instrument that measures dispositional forgiveness of self, others, and situations. In the first study, 203 students (65% women) participated. After ensuring the linguistic adequacy and clarity of the wording of the items, a lack of congruence was found between the factors obtained in the exploratory factor analysis and the original theoretical structure of the HFS. A sample of 512 participants (63.9% women) attended the second study. This study aimed to analyze the construct validity of the HFS using confirmatory factor analysis through structural equation modelling and to explore convergent, discriminant, and criterion validity. Of the different factorial configurations tested (including the original), only a scale reduction to eight items, grouped into three factors, showed an appropriate fit. The HFS eight-item version also showed acceptable internal consistency, adequate convergent and discriminant validity, and criterion validity with respect to related variables. These findings suggest that the eight-item version of the HFS may be a valid and reliable tool for assessing forgiveness for self, others, and situations in Spanish adults.

## 1. Introduction

For over 20 years, the notion of forgiveness has been a subject of study within the social sciences. Understood as a multidimensional concept, forgiveness is a process which allows individuals to overcome the negative psychological consequences of being wrongfully harmed [1,2]. The process begins when the victim of aggression becomes aware they have been harmed by another [3]. Within this process, the victim experiences a diminishment of negative thoughts, emotions, and motivations towards their aggressor, with an increase in positive thoughts, emotions, and motivations towards this person [2,4,5].

Numerous studies have found associations between forgiveness and a number of indicators of mental health and psychological wellbeing. It has been associated with high self-esteem, higher levels of psychological wellbeing [6,7,8], better conflict management [9], greater satisfaction in close relationships [10], lower levels of narcissism [11], and greater spiritual wellbeing [12,13]. Forgiveness correlates positively with overall mental health [14], quality of relationships [14], hope [15], and existential and spiritual wellbeing [15], with a negative correlation to depression [12], stress [14], anger [14,15], and anxiety [16].

Forgiveness must be clearly differentiated and not confused with other terms, such as reconciliation or the desire for reconciliation. According to Enright, Gassin, and Wu [17], “forgiveness is an internal liberation and concerns an individual. Reconciliation, on the other hand, involves the interaction between two parties” (p. 104). Although these terms are commonly confused among the general public, doing so would mean assuming forgiveness as a process to resume and repair relationships [18] conditioned by another and setting aside one’s individuality of the process.

Different classifications of forgiveness have been proposed. One is determined by who receives it and who is offended; in this sense, Enright [19] proposes the so-called “triad of forgiveness”, consisting of the forgiveness of others, forgiveness of self, and receiving forgiveness. In 2005, Thompson [20] and his collaborators added forgiveness of situations. This type of forgiveness arises when there is no clear offender, such as a disease or natural disaster [20]. However, this latter distinction has caused controversy and opposing positions among researchers, some of whom argue that people forgive others and not situations [21]. The authors [20], however, argue that forgiveness of situations is related to forgiveness as a general concept, and differs from forgiveness of self and others. Although intentionality is an important factor in the process of forgiveness, and situations themselves are unintentional, people often blame or forgive others even while recognizing the harm was unintentional. Thus, evaluating only forgiveness of self and others may neglect the contribution of further aspects of forgiveness [20]. 

Another classification of the notion of forgiveness refers to whether it is specific, that is, occurring after and referring to a specific act of harm or aggression, or, rather, a general tendency of an individual to forgive others, given that some people are more likely to forgive than others. The first distinction refers to specific forgiveness, forgiveness of a particular person or offense. In the second case, there is talk of dispositional forgiveness, a general tendency of people to forgive, analogous to a personality trait [22].

A large number of instruments are available to measure dispositional forgiveness [12,15,20,23,24,25]. However, the scale developed by Laura Thompson and her collaborators in 2005, called the Heartland Forgiveness Scale (HFS), is one of the most widely-used, and is unique in measuring forgiveness of self, others, and situations. This scale has been translated many times, even by the authors themselves, which are freely available on their website [26].

The HFS was developed to measure dispositional forgiveness in a multidimensional sense; in the process of constructing the instrument, the authors initially proposed that dispositional forgiveness consists of three interrelated factors (forgiveness of self, forgiveness of others, and forgiveness of situations) with six items in each of these three subscales. However, this model did not have an adequate fit [20], and the authors proposed an alternative and more complex bifactor structure with six factors (with three items each). In this second structure, each of the three forgiveness dimensions (forgiveness of oneself, forgiveness of others, and forgiveness of circumstances), was subdivided into two factors with three items each (the one with those positively worded items and the other with those negatively worded items), thus resulting in six factors. In addition, the three factors with positively worded items loaded in a latent variable, whereas the three factors with negatively worded items loaded in another different latent variable. This more complex alternative obtained an excellent fit [20].

This scale has been translated into over twenty languages and has been subjected to adaptation and validation studies for different populations; for example, for the Turkish [27], Indian [28], Taiwanese [29], and, more recently, the Portuguese [30]. Although Spanish translations of the scale are available and have been used in some research projects [31,32], there is currently no study of the adaptation and validation for the Spanish population. Therefore, the aim of this work is to culturally adapt and validate the HFS to the Spanish population. 

## 2. Materials and Methods 

The two studies conducted for this research are presented below. The first was a pilot study describing the process of cultural adaptation of HFS, with an exploratory analysis of the factor structure and the HFS psychometric properties. The second study analyses both the construct validity of the HFS through confirmatory factor analysis (CFA) using structural equation methodology and the reliability and convergent, discriminant, and criterion validity. 

### 2.1. Study 1: Pilot Study

Study 1 consists of a pilot study whose objective is to culturally adapt the HFS, preliminarily explore its psychometric properties (descriptive statistics, normality, and reliability) and analyze its factor structure in an exploratory manner.

#### 2.1.1. Participants 

The study was conducted using a sample of 203 participants, of whom 65% were women (*n* = 132) and 35% male (*n* = 71), between the ages of 18 to 28 (mean (M) = 20.5 years; standard deviation (SD) = 2.3). All participants were university students from the Community of Madrid and Spanish nationals. Of the total participants, 98.5% were single and 1.5% were married.

Non-probabilistic sampling was used to contact the participants. University students in different degree programs (psychology, education, and physiology) were invited to take part in the study. Participants responded to the questionnaire using pencil and paper. After signing the informed consent, they were asked to read the questions and rate their answers on the appropriate scale. The confidentiality of this data was safeguarded in accordance with Spanish Law (Organic Law 5/2018 on Data Protection and Guarantee of Digital Rights), and the ethical principles of the Helsinki Declaration were followed.

#### 2.1.2. Instruments

Sociodemographic questionnaire: An ad hoc instrument developed for this research collecting the following information from the participants: gender, age, nationality, and education level. 

Heartland Forgiveness Scale (HFS) [20]: An 18-item self-reported instrument that measures dispositional forgiveness. It consists of three subscales of six items each: (a) forgiveness of self: items 1 to 6; (b) forgiveness of others: items 7 through 12; and (c) forgiveness of situations: items 13–18. Participants are asked to indicate the degree to which they identify with each sentence using a seven-point Likert scale (1 = almost always false for me, to 7 = almost always true for me). A higher score reflects an individual’s greater willingness to forgive others, himself (or herself), and/or situations, and vice versa. The authors report adequate internal consistency, Cronbach’s alpha, with values between 0.72 and 0.87 [20] and test–retest reliability for a 3-week interval of 0.72–0.77 and 9 months of 0.68–0.69 [20]. In some previous studies, internal consistency values between 0.48 and 0.86 were found for the different subscales [27,30,31]. In the present study, an internal consistency of 0.81 was obtained for the total scale, while the reliability values of the different subscales ranged between 0.67 and 0.79. 

As this was the first adaptation of the HFS to the Spanish population, the research followed the guidelines of the International Test Commission for cross-cultural translation and adaptation of instruments by Muñiz, Elosua, and Hambleton [33] and Gjersing et al. [34]. Some of the recommendations of the American Educational Research Association, the American Psychological Association and the National Council on Measurement in Education [33,34] were also followed.

First, a panel of experts in forgiveness psychology was consulted to confirm the relevance of an adaptation of the scale. Second, permission was obtained to translate and adapt the instrument from Laura Thompson, holder of the intellectual property rights of the instrument. With this permission and a previous Spanish translation of the scale by the author (available on her website), two bilingual psychologists, familiar with the Spanish culture, reviewed the Spanish version to ensure it was culturally appropriate. After two revisions, the differences between the two versions were corrected with the help of a third researcher. The panel of experts reviewed the final version and confirmed the adequacy of the linguistic and cultural aspects of the items for Spanish culture.

In order to assess the clarity and exactness of the measure and the comprehensibility and difficulty of the items, the scale was evaluated by 23 students (56.5% women) between 20 and 80 years of age (M = 30.13 years; SD = 14.46) with heterogeneous sociodemographic characteristics. The majority of participants had postgraduate university studies (43.5%), followed by those with a Bachelor’s degree (30.45%), high school (17.5%) or secondary studies (4.3%), and primary education (4.3%). The participants proposed minor linguistic modifications to 9 of the 18 items, which did not alter the meaning of the original version. The final version of the instrument is provided in the supplementary material (Supplementary A).

#### 2.1.3. Data Analysis

The statistical package for social sciences IBM SPSS for Windows version 22.0 (Armonk, NY, USA) was used to analyze the data. The following analyses were performed: descriptive analyses, including asymmetry and kurtosis, with values between −2 and 2 as indicative of univariate normality [35]; and reliability analysis, considering values equal or above 0.70 in Cronbach’s alpha as appropriate. Given the data characteristics and the theoretical basis of the instrument, exploratory factor analysis (EFA), using principal components analysis method (PC) with Promax rotation, was conducted for factor structure detection. Factors with eigenvalues greater than 1 were extracted. In addition, sample adequacy was assessed by the Kaiser–Meyer–Olkin (KMO) (with values above 0.60 indicating adequacy), and the sufficiency of the model was determined with Bartlett’s sphericity test (with significant values of the χ^2^ statistic suggesting the factorability of the correlation matrix) [36,37]. The criterion for the inclusion of items was a factor loading over 0.30 [38,39], and a minimum value of 0.40 was established for the item’s communalities [40].

### 2.2. Results

The descriptive statistics of the HFS items are provided in Table 1. 

The HFS, as a whole, shows an internal consistency of 0.81, and the following reliability indices of each subscale were obtained using Cronbach’s alpha: 0.70 for forgiveness of self, 0.67 for forgiveness of others, and 0.79 for forgiveness of situations.

With regard to the factor structure, the suitability index of the sample using the KMO showed the adequacy of the data (KMO = 0.80). Bartlett’s sphericity test was significant for the scale (χ^2^ = 1084.895; df = 171, *p* < 0.001). Using the PC method, a factorial solution without restricting the number of factors showed four factors that accounted for 53.79% of the variance. With the exception of items 3 and 12 (0.34 and 0.39, respectively), all elements showed communalities between 0.40 (item 8) and 0.72 (item 17). The matrix was analyzed using Promax rotation; the grouping trends of the items are shown in Table 2.

The rotated matrix restricted to three factors was then explored, according to the number of HFS subscales of the original version. This matrix explains 47.86% of the variance and, as shown in Table 2, several of the items did not reach the communality value of 0.40. The factor matrix identifies the three dimensions, but not clearly, finding certain items that loaded simultaneously on more than one factor.

### 2.3. Study 2: Validity Study 

The aim of this study was to analyze the construct validity of the HFS using confirmatory factor analysis (CFA) through structural equation modelling (SEM) and to explore its convergent, discriminant, and criterion validity through correlations with other relevant variables.

#### 2.3.1. Participants

A total of 512 adults from the general population participated in the study (sociodemographic characteristics are summarized in Table 3).

Participants were selected through non-probabilistic sampling. The Google Forms platform was used to disseminate the instruments, which made it possible to ensure that they were fully completed and there were no missing values. The inclusion criteria for participants were: (a) to be over 18 years of age, and (b) to sign the informed consent form for participation in the study pursuant to Organic Law 5/2018 on Data Protection and Guarantee of Digital Rights. The ethical guidelines of the Helsinki Declaration were also followed.

#### 2.3.2. Instruments

Sociodemographic questionnaire: Used and described in the pilot study. 

Heartland Forgiveness Scale (HFS) [20]: Translated version adapted and described in the pilot study. 

Explicit Self-Forgiveness Item [8]: Item in which the participant is asked to respond to the following statement: “When I consider what I did to be wrong, to what extent I think I have forgiven myself”, responding on a five-point Likert scale ranging from “not at all” to “completely”. This item has generally been used as a measure of validity of the State Self-Forgiveness Scale (SSFS) of Wohl et al. [8]. It has been found that those obtaining high scores show high degrees of feelings, actions, and behaviors of self-forgiveness [8].

Acceptance of Responsibility Scale [41]; translated into Spanish for this research: This instrument consists of eight items used to measure the admission of responsibility by the offender, understood from a moral sense: recognizing wrongful behavior, the seriousness of the action, the lack of justification, and acceptance of guilt. Participants respond to each item based on a situation in which they recall having acted wrongfully. The instrument uses a Likert type scale from 1 (totally disagree) to 7 (fully agree). Some examples of the items are “I feel responsible for what happened” or “I wasn’t really to blame for this”. The authors found a high internal consistency (α = 0.91), similar to that obtained in the study (α = 0.83).

Desire for Reconciliation Scale [42]; translated into Spanish for this research: This instrument consists of five items used to measure the desire for reconciliation of those who have acted wrongfully. It includes items such as “I want to be reconciled with this person” and “I want the relationship between this person and me to improve.” Participants respond using a seven-point Likert scale from 1 (I don’t agree at all) to 7 (totally agree). High scores on the scale indicate a greater intention to repair the relationship with those whom you have wronged or offended. Woodyatt and Wenzel [42] provide evidence of adequate internal consistency in their study (α = 0.82); in the current sample, Cronbach’s alpha was 0.86.

Mental Health Inventory (MHI-5) [43]; validation of the Spanish version by Alonso, Prieto & Antó [44]: This is a scale of five items that evaluates the participant’s overall mental health based on the level of anxious and depressive symptoms in the last month. Participants respond using a six-point Likert scale from 1 (never) to 6 (always). In the Spanish translation, Alonso et al. [44] reported an internal consistency of 0.77. In the present study, Cronbach’s alpha of 0.89 was found.

Psychological Well-Being Scale (PWBS [45]; Spanish adaptation by Díaz et al. [46]): This is a scale that aims to provide a reliable measure of psychological wellbeing understood from a eudaimonic perspective. The questionnaire assesses six wellbeing dimensions: self-acceptance, positive relationships with others, autonomy, environment mastery, purpose in life, and personal growth. The present study used a condensed version of the original scales [46], consisting of 29 items with a 6-point Likert scale (1 = totally disagree to 6 = totally agree). Some examples of the items are “I’m not afraid to express my opinions, even when they’re contrary to those of most people” or “I’m worried about how other people judge the choices I’ve made in my life”. Each of the six dimensions showed an internal consistency above 0.70 [46]. In this study, the reliability of the total score was high (α = 0.82), and the Cronbach’s alpha values for the subscales ranged from 0.70 (autonomy) to 0.85 (purpose in life). Given that the aim of this research is to determine psychological wellbeing as a whole, the total score was used.

#### 2.3.3. Data Analysis 

CFA was performed to test the adequacy of both the structure proposed by the authors of the original scale and other alternative structures. Using EQS for Windows version 6.2 (Encino, CA, USA), an SEM analysis was made using the robust maximum likelihood estimation method, due to the non-normality of the data suggested by a Mardia’s standardized coefficient above three. The goodness of fit of the assessed models was evaluated by: (a) the Satorra–Bentler (S–B) χ^2^, its degrees of freedom (df), and *p* value; (b) the comparative fit index (CFI), as an incremental fit index; and the (c) the root mean square error of approximation (RMSEA) with its 90% confidence interval (CI). Adequate model fit was determined by the following cutoff: S–B χ^2^ *p* value ≥ 0.05, CFI ≥ 0.92, and RMSEA ≤ 0.07 [47]. Given that large sample sizes can negatively affect the interpretation of the S–B χ^2^ statistic, it is preferable to use the S–B χ^2^/df ratio, where values between one and three are indicative of good adjustment [47]. Additionally, to verify the adequacy of the different SEM models, the study explored the absence of improper solutions (parameters and values that are logically and mathematically impossible), such as negative or nonsignificant error variances, nonpositive definite correlation matrix, or out-of-range parameters. These problems may indicate multicollinearity, the presence of outliers, or a misspecification of the model, among others, which could require the elimination of indicators (items) or the respecification of the model itself [48].

The CFA-based reliability was also tested by calculating composite reliability (CR), given that in SEM, Cronbach’s alpha can overestimate or underestimate the true reliability [48]. CR values greater than or equal to 0.70 are considered good [47].

Finally, using the SPSS program, the Pearson’s correlation coefficient was calculated to determine convergent and discriminant validity and criterion validity.

### 2.4. Results

#### 2.4.1. Factor Structure

**Figure 1:**
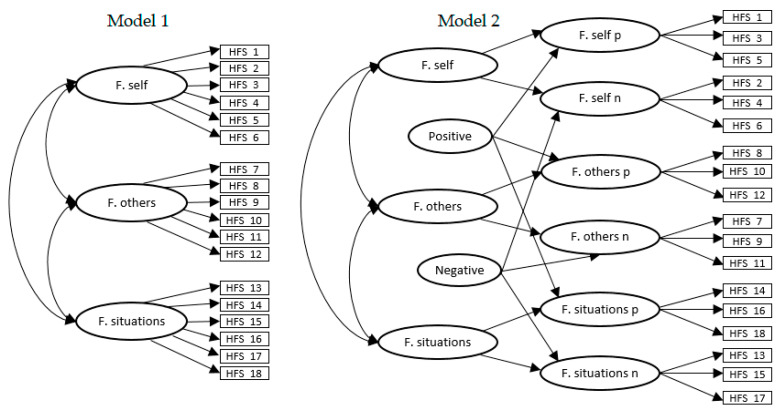
Model 1 and model 2. F. self = forgiveness of self; F. others = forgiveness of others; F. situations = forgiveness of situations; p = positively worded items; n = negatively worded items.

Based on the initial proposal of the authors [20], in model 1 (see Figure 1), the 18 items of the HFS were grouped into three subscales of six elements each (three positively worded and three negatively worded): the forgiveness of self subscale was made up of items 1, 3, and 5 (positively worded) and items 2, 4, and 6 (negatively worded); the forgiveness to others subscale was composed of items 8, 10, and 12 (positively worded) and of items 7, 9, and 11 (negatively worded); and the subscale forgiveness of circumstances grouped items 14, 15, and 16 (positively worded) and items 13, 15, and 17 (negatively worded). The fit indices of this structural model showed poor adequacy (see Table 4).

Subsequently, the aforementioned complex bifactor structure with six factors, proposed by the authors [20] of the original version, was then tested (model 2; see Figure 1). Despite the good fit obtained (Table 4), the model proved unsatisfactory as improper solutions were found, specifically, negative error variances and paths that could not be estimated.

**Figure 2:**
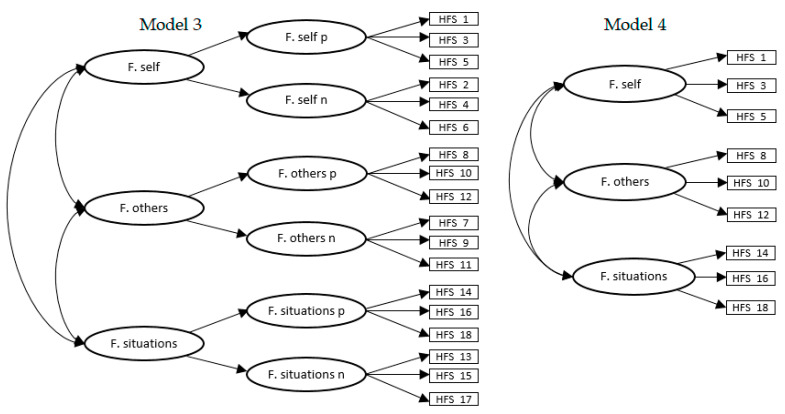
Model 3 and model 4. F. self = forgiveness of self; F. others = forgiveness of others; F. situations = forgiveness of situations; p = positively worded items; n = negatively worded items.

Thirdly, to explore whether a simplification of the model could eliminate the presence of improper solutions, the same previous model was tested, but eliminating positive and negative latent factors (model 3; see Figure 2). The 18 items grouped into six factors were maintained in six factors (forgiveness of self, positive and negative; forgiveness of others, positive and negative; and forgiveness of situations, positive and negative) which were simultaneously grouped into three higher order factors. Despite the adequate adjustment indices obtained (Table 4), the model was unsatisfactory, as negative variances, a nonpositive definite correlation matrix, and out-of-range parameters were found.

A fourth model (model 4; see Figure 2), more abbreviated, was tested, in which only the positive wording items were included. Thus, in this model, dispositional forgiveness was made up of nine positive items grouped into three correlated factors. However, despite the good fit and the absence of improper solutions (Table 4), the model proved unsatisfactory due to the low reliability coefficients obtained in two of the three dimensions (0.62 and 0.52 for forgiveness of self and forgiveness of others, respectively).

**Figure 3:**
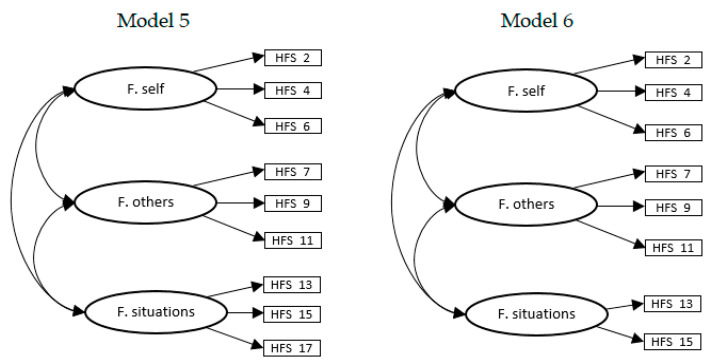
Model 5 and model 6. F. self = forgiveness of self; F. others = forgiveness of others; F. situations = forgiveness of situations.

A fifth abbreviated model was tested (model 5; see Figure 3), in this case consisting of 9 negative wording items grouped into three correlated factors (forgiveness of self, forgiveness of others and forgiveness of situations). Although good adjustment indices were obtained (Table 4), the model proved unsatisfactory showing both an improper solution with nonsignificant variance estimates and a factor loading equal to 1.

Finally, a model almost identical to the previous one was analyzed, in which the last item of the forgiveness of circumstances factor was eliminated (model 6; see Figure 3), due to the problems of improper solutions discussed in the previous model. In this model, composed of the remaining eight negative wording items, the items are grouped into three related factors (with items 2, 4, and 6 in the forgiveness of self; items 7, 9, and 11 in the forgiveness to others; and items 13 and 15 in the forgiveness of situations). Good reliability (CR) was also observed in the factors forgiveness of self (0.78) and forgiveness of situations (0.87), and acceptable for forgiveness of others (0.62). It was decided to remove item 17 because (as indicated by its high standardized residuals and the presence of standardized factor loadings of 1 in this last dimension) it also seems to correlate with other subscales of the instrument to which it does not belong. Additionally, at a semantic level, the item is less specific than the other two on the subscale: item 17 states “it is difficult to accept uncontrollable situations”, whereas the other two items specify that it is difficult to stop engaging in negative thoughts due to uncontrollable situations. In contrast to the problems found in the previously analyzed models, the factor structure tested in model 6 yielded satisfactory results, as it showed good fit indices (Table 4) and an absence of improper solutions.

#### 2.4.2. Convergent, Discriminant, and Criterion Validity 

Regarding convergent validity, as shown in Table 5, the scale showed moderate, positive, and significant associations with the variables mental health and psychological wellbeing. In terms of discriminant validity, as expected, no significant correlation was found with the desire for reconciliation (see Table 5). Finally, regarding criterion validity, positive and significant correlations were found (r = 0.40; *p* < 0.01; M = 3.5; SD = 1.16) between the HFS and another measure of forgiveness of self (explicit self-forgiveness item [8]).

## 3. Discussion

The aim of this study was to validate the HFS for the Spanish population. Although some research, such as by McConnell, Dixon, and Finch [49], Strelan [50], or Rangganadhan and Todorov [51], carried out mainly among Australian and American populations, showed that the instrument appeared to assess dispositional forgiveness effectively, the results of this study suggest that, when used for the Spanish population, the instrument may suffer reliability and validity problems if no modifications are made. In fact, research by Prieto-Ursúa et al. [31] pointed to certain drawbacks in the reliability of the instrument, reporting a Cronbach’s alpha of 0.60 for the subscale of forgiveness of self and 0.48 for forgiveness of others. Thus, it is important that studies such as this one explore in depth the psychometric properties of the HFS in different groups and samples, especially due to its wide use as a measure of dispositional forgiveness (of self, others, and situations).

Regarding the EFA, the results without restricting factors showed a fourth element that was not consistent with the theory proposed by the authors; however, by forcing the restriction of the factors to three factors, as suggested by the original model, an acceptable percentage of explained variance was obtained. The CFA showed that the complete version of the instrument does not perform satisfactorily for the Spanish population. This led to the exploration of other alternative factor structures. Among them, a reduced version of the instrument made up of eight items was the one that showed an adequate fit and an absence of mathematical incongruences (i.e., improper solutions). According to this model, the measure of dispositional forgiveness consists of eight negative items and three interrelated factors: forgiveness of self, forgiveness of others, and forgiveness of situations. In this version, each factor shows acceptable indicators of reliability (0.78 in forgiveness of self, 0.87 in forgiveness of situations, and 0.62 in forgiveness of others).

In this brief version of the HFS, the subscale of forgiveness of situations consists of two, rather than three, items, as in the other subscales. Beyond the fact that psychometric analyses show this abbreviation makes the instrument more reliable, we believe that retaining this item can cause confusion due to semantic reasons, as the deleted statement was very general and ambiguous. Using only the two remaining items, the person who responds is contextualized and placed in a situation which allows greater emphasis on the controllability or intentionality of the situations, a fact that, according to the authors of the original scale, acquires great value in the process of forgiveness [20]. 

Regarding the exploration of convergent and discriminant validity, the results of this study are in line with previous research, and show that psychological wellbeing and mental health are significantly related to dispositional forgiveness [6,7,8,14], as well as that forgiveness is conceptually different from the desire for reconciliation. Thus, this reinforces the notion that forgiveness should not be understood as a process for the reestablishment of a relationship between the victim and aggressor [17,18]. In terms of criterion validity, the dimension of the HFS forgiveness of self was significantly associated with the single item of forgiveness to self. These findings support that the eight-item brief version of the HFS may be an instrument for assessing dispositional forgiveness (to self, to others, and to situations) with sufficient reliability and validity to be used in the Spanish population.

Furthermore, it is necessary to continue generating research in this area of study, deepening the concept of forgiveness and the development of valid and reliable instruments. Specifically, it would be interesting if future studies could focus on the validation in the Spanish population of other widely-used forgiveness measures, such as Woodyatt and Wenzel’s Differentiated Self-Pardoning Process Scale [52] or the Enright Forgiveness Inventory (EFI) [53]. In addition, it is recommended that research be extended to other, less -explored age ranges, such as youth and older adults.

The present study has certain limitations. First, although large and relatively heterogeneous samples were used, a representative sampling was not carried out, which may affect the external validity of the study. Since the scale used is a self-reported instrument, it is possible that the results were influenced by the social desirability bias. Furthermore, it should be mentioned that the instruments to measure the desire for reconciliation and the acceptance of responsibility have not been previously validated in the Spanish population. Finally, as our study is part of a broader project focused on forgiveness of self, we provided data on the validity of criteria only for the subscale of forgiveness of self, and not for the subscale of forgiveness of others (for the subscale of forgiveness of situations, we are unaware of any instruments to verify this correlation), so future studies should address this issue.

## 4. Conclusions

The study presents interesting findings. Our results indicate the HFS should be adapted for its application in the Spanish population, especially in order to maintain the factorial structure proposed by the original authors. For this reason, this adaptation of the original scale is proposed: an abbreviated version of eight items that should continue to be tested in future research. Despite the fact that the structure of the original instrument has not been kept invariant, and a large number of items have been removed, it is very interesting to continue maintaining the subscale of forgiveness of situations as part of the measure of forgiveness. In this vein, the HFS is currently the only scale adapted and validated in the Spanish population that allows to simultaneously measure dispositional forgiveness of oneself, others, and situations. The HFS can be a very useful instrument both for psychological research and for use in clinical and health settings. In addition, its great brevity makes it a test of easy and quick application in a wide variety of contexts. 

## Figures and Tables

**Table 1:** Heartland Forgiveness Scale (HFS) descriptive statistics in the pilot study (*n* = 203).

Item	M	SD	Asymmetry	Kurtosis
HFS 1	4.81	1.57	−0.52	−0.18
HFS 2	4.07	1.77	0.03	−0.93
HFS 3	6.00	1.32	−1.09	0.83
HFS 4	4.23	1.69	−0.11	−0.87
HFS 5	5.84	1.15	−0.95	0.63
HFS 6	4.27	1.67	−0.08	−0.79
HFS 7	5.41	1.48	−0.87	0.38
HFS 8	5.58	1.26	−0.79	0.45
HFS 9	6.10	1.33	−1.47	1.34
HFS 10	4.84	1.77	−0.54	−0.76
HFS 11	3.70	1.78	−0.21	−0.89
HFS 12	5.15	1.50	−0.76	0.20
HFS 13	4.13	1.62	−0.02	−0.72
HFS 14	5.26	1.24	−0.52	−0.08
HFS 15	4.27	1.69	0.06	−0.97
HFS 16	4.45	1.64	−0.43	−0.44
HFS 17	4.66	1.70	−0.27	−0.81
HFS 18	4.87	1.62	−0.49	−0.50

*M* = Mean; *SD* = Standard deviation.

**Table 2:** Rotated factor matrix of the HFS with restricted and unrestricted solutions in the pilot study (*n* = 203).

Unrestricted						Restricted to 3 Factors				
Item	Factor				Communality	Item	Factor			Communality
	1	2	3	4			1	2	3	
HFS 2	0.87				0.68	HFS 4	0.76			0.54
HFS 4	0.82				0.61	HFS 6	0.74			0.62
HFS 6	0.76				0.67	HFS 2	0.71			0.55
HFS 5	−0.39	0.92			0.62	HFS 13	0.69			0.55
HFS 1		0.58			0.42	HFS 15	0.67			0.60
HFS 3		0.53			0.34	HFS 17	0.60			0.38
HFS 18		0.52			0.40	HFS 5		0.83		0.56
HFS 14		0.52			0.55	HFS 16		0.57		0.49
HFS 16	0.41	0.49			0.55	HFS 14		0.55		0.54
HFS 9			0.79		0.59	HFS 1		0.54		0.41
HFS 7			0.75		0.53	HFS 18		0.49		0.39
HFS 11			0.69		0.42	HFS 3		0.44		0.29
HFS 8	−0.37		0.47		0.40	HFS 10		0.40	0.39	0.38
HFS 10			0.46	−0.37	0.47	HFS 12		0.38	0.35	0.34
HFS 12			0.40		0.39	HFS 9			0.78	0.59
HFS 17				0.85	0.72	HFS 7			0.72	0.52
HFS 13				0.56	0.60	HFS 11			0.67	0.42
HFS 15	0.35			0.44	0.67	HFS 8			0.48	0.39

Extraction method: principal components analysis. Rotation method: promax with Kaiser normalization.

**Table 3:** Sociodemographic characteristics of the participants of the validity study (*n* = 512).

Age [18–67 Years], *Mean*, (*SD*)	Characteristics	38.5	(13.48)
		*n*	%
Gender	Male	185	36.1
	Female	327	63.9
Civil status	Single	217	42.4
	Common-law	42	8.2
	Married	197	38.5
	Divorced	26	5.1
	Separated	7	1.4
	Widowed	5	1
	Other	18	3.5
Education level	Primary	1	0.2
	Secondary	19	3.7
	High School	94	18.4
	Bachelor’s degree	191	37.3
	Postgraduate degree	204	39.8
	Other	3	0.6

*M* = Mean; *SD* = Standard deviation.

**Table 4:** Goodness of fit indexes for the models assessed in the validity study.

Models	S–B χ^2^	df	*p*	CFI	RMSEA	RMSEA 90% CI	S–B χ^2^/df Ratio
Model 1. 3-factor and 1st order 18-item structure (positive and negative items)	1527.958	132	<0.001	0.674	0.144	[0.137–0.150]	11.575
Model 2. 6-factor bifactor 18-item structure (positive and negative items)	308.684	121	<0.001	0.956	0.055	[0.047–0.063]	2.551
Model 3. 6-factor and 2nd order 18-item structure (positive and negative items)	387.717	126	<0.001	0.939	0.064	[0.057–0.071]	3.077
Model 4. 3-factor and 1st order 9-item structure (positive items)	79.413	24	<0.001	0.957	0.067	[0.051–0.084]	3.309
Model 5. 3-factor and 1st order 9-item structure (negative items)	52.365	24	<0.001	0.990	0.048	[0.030–0.066]	2.182
Model 6. 3-factor and 1st order 8-item structure (positive items)	33.874	17	0.009	0.986	0.044	[0.022–0.066]	1.993

S–B χ^2^ = Satorra–Bentler scaled Chi square statistic; df = degrees of freedom; CFI = comparative fit index; RMSEA = root mean square error of approximation; RMSEA 90% CI = RMSEA 90% confidence interval; S–B χ^2^/df ratio = S–B χ^2^ divided by df.

**Table 5:** Descriptive statistics and correlations (r) between HFS and psychological wellbeing, mental health, and desire for reconciliation obtained in the validity study (*n* = 512).

Variable	r	M	SD
Psychological wellbeing	0.61 **	4.62	0.69
Mental health	0.58 **	3.46	0.88
Desire for reconciliation	0.01	23.63	5.88

** *p* < 0.01.

## Data Availability

The data presented in this study are available on request from the corresponding author. The data are not publicly available due to because it belongs to a doctoral thesis research that is still in progress.

## Supplementary Materials

Supplementary A (Spanish Version of the HFS used in the study) is available online at https://www.mdpi.com/1660-4601/18/1/45/s1.
