# Supplementary material for: Measuring Forgiveness: Psychometric Properties of the Heartland Forgiveness Scale in the Spanish Population
Source: Int J Environ Res Public Health. 2020 Dec 23;18(1):45. doi: 10.3390/ijerph18010045 (PMC7793486; doi:10.3390/ijerph18010045)
Supplement: Supplementary file 1 [file ijerph-18-00045-s001.pdf]

Supplementary A: Version of the HFS used in the study (Spanish version)

En el curso de nuestra vida pueden surgir eventos negativos como resultado de nuestra propia acción, las acciones de otro/s, o por Circunstancias fuera de nuestro control. Durante algún tiempo después del evento, puede que aún guardemos sentimientos o pensamientos negativos sobre nosotros mismos, las otras personas o la situación. Piense sobre cómo típicamente usted reacciona a tales eventos negativos. En el espacio que sigue a cada frase, marque (según la escala de 7 puntos abajo), el número que más acertadamente describe cómo típicamente usted responde a la situación negativa descrita. No hay respuestas correctas o incorrectas. Por favor, sea lo más sincero/a posible con sus respuestas.

1	2	3	4	5	6	7
Casi siempre falso para mí		Más bien falso para mí		Más bien cierto para mí		Casi siempre cierto para mí

Ítems de la escala
1. Aunque al principio me siento mal cuando me equivoco, con el tiempo puedo darme algún respiro.
**2. Me guardo rencor a mí mismo/a por las cosas negativas que he hecho.**   **(I hold grudges against myself for negative things I've done).**
3. Aprender de las cosas malas que he hecho me ayuda a superarlas.
**4. Me resulta realmente difícil aceptarme cuando he cometido un error.**   **(It is really hard for me to accept myself once I've messed up).**
5. Con el tiempo voy comprendiendo los errores que he cometido.
**6. No dejo de criticarme por las cosas negativas que he sentido, pensado, dicho o hecho.**   **(I don't stop criticizing myself for negative things I've felt, thought, said, or done).**
**7. No dejo de castigar a alguien que ha hecho algo que creo que es incorrecto.**   **(I continue to punish a person who has done something that I think is wrong).**
8. Con el tiempo, llego a ser comprensivo/a con otros por errores que hayan cometido.
**9. Sigo haciéndole la vida difícil a las personas que me hayan herido.**   **(I continue to be hard on others who have hurt me).**
10. Aunque otros me hayan hecho daño en el pasado, he podido llegar a verles como buenas personas.
**11. Me quedo pensando mal de quien me haya maltratado.**   **(If others mistreat me, I continue to think badly of them).**
12. Cuando alguien me decepciona puedo llegar a pasar página.
**13. Cuando suceden cosas malas por razones que no se pueden controlar, me quedo estancado/a en pensamientos negativos sobre el evento.**   **(When things go wrong for reasons that can't be controlled, I get stuck in negative thoughts about it).**
14. Pasado un tiempo suelo ser comprensivo con las circunstancias negativas en mi vida.
**15. Si me siento decepcionado/a por circunstancias incontrolables en mi vida, no puedo dejar de pensar negativamente en ellas.**   **(If I am disappointed by uncontrollable circumstances in my life, I continue to think negatively about them).**
16. Al final me encuentro en paz con las circunstancias negativas de mi vida.
17. Se me hace muy difícil aceptar circunstancias negativas que no son culpa de nadie.
18. Al final, puedo dejar atrás pensamientos negativos sobre circunstancias malas que nadie puede controlar.

Note. The sentences in bold correspond to those items included in the eight-item version of the scale that have shown adequate psychometric properties in the Spanish population. For each of the eight items proposed in

this Spanish version, the wording by Laura Y. Thompson (https://www.heartlandforgiveness.com/) in its original version of 18 items has been included in parentheses.
